# Antiferromagnetic Spin Wave Amplification by Scattering in the Presence of Non-Uniform Dzyaloshinskii–Moriya Interaction

**DOI:** 10.3390/ma17225585

**Published:** 2024-11-15

**Authors:** Taeheon Kim, Geun-Ju Kim, Jung-Il Kim, Kwang-Ho Jang

**Affiliations:** Electro-Medical Equipment Research Division, Applied Electromagnetic Wave Research Center, Korea Electrotechnology Research Institute, Ansan 15588, Republic of Korea; gjkim@keri.re.kr (G.-J.K.); sky@keri.re.kr (J.-I.K.); khjang@keri.re.kr (K.-H.J.)

**Keywords:** antiferromagnet, terahertz frequency, Dzyaloshinskii–Moriya interaction

## Abstract

In this study, we suggest a method to amplify spin waves (SWs) in antiferromagnets (AFMs). By introducing a non-uniform Dzyaloshinskii–Moriya (DM) interaction, the potential barrier forms a resonant cavity. SWs with an opposite chirality undergo scattering and are resonantly amplified at a phase-matching condition. The calculation is performed in the insulating AFMs where the electric-field-induced DM interaction and pseudo-dipole anisotropy broaden the parabolic-like SW band for multiple resonant modes. Using a transfer matrix method, we also show numerically that scattering between SWs contributes significantly to the SW amplification. Since the electric field selectively amplifies the SWs with resonant frequencies, the proposed device works as an SW transistor and rectifier. This finding will contribute to insulating AFM-based magnon devices where Joule heating is, in principle, avoided.

## 1. Introduction

Spin waves (SWs) are collective excitations in magnetically ordered media, which are called magnon because of their particle-like nature. Since SW carries information similar to what the spin current does without a charge flow, it can be a good candidate for low-dissipation devices in magnon spintronics [1,2,3].

SW has precessional polarizations, similar to electromagnetic waves. A ferromagnet (FM) has only a single circularly polarized mode due to broken time-reversal symmetry, while antiferromagnetic SWs have two circularly polarized modes due to the negative exchange interaction. Using these properties, the phases of antiferromagnetic SWs have been controlled [4,5,6], including manipulating the polarization [6,7,8] in the system with a lack of inversion symmetry. Such AFM SWs, depending on two different polarizations, are detectable via the inverse spin Hall effect [9,10] and can be utilized with magnon torques for magnetization switching [11]. Thus, AFM-based magnonics are promising compared to their ferromagnetic counterparts.

Despite the advantages of AFM spin waves, a major challenge lies in extending their propagation length within a medium. A large amplitude for a given input power enables stable and robust wave propagation over a long distance. One approach to overcome this challenge is the use of low-damping materials, a strategy that has been extensively studied [12,13,14,15]; however, this method is fundamentally constrained by the intrinsic properties of the materials themselves. Another approach focuses on extrinsically modifying the propagation characteristics of SWs. It has been demonstrated in FMs and AFMs that a current-induced spin-transfer torque (STT) can amplify SWs, altering their attenuation length [16] and damping constant [17]. Through SW scattering [18], the SW dispersion relations shift due to the Doppler effect [19], which enables resonant amplification of emerging SWs. However, SW scattering has been primarily implemented in FMs and has not yet been established in AFMs. The realization of SW scattering by Doppler shift in AFMs seems to be difficult compared to that in FMs [18]; the equations of motion of AFMs are the second order in frequency rather than the first order of FMs. This means that the negative frequencies that play a key role in resonant scattering in FMs [18] are not manipulated to the positive ones by an SW Doppler shift in AFMs.

Recognizing that implementing a Doppler shift in AFMs is not feasible, we propose an alternative method to amplify antiferromagnetic SWs. This method leverages the Dzyaloshinskii-Moriya (DM) interaction and pseudo-dipole anisotropy induced by an electric field. Our target materials are Mott insulating AFMs, which are known to induce both the DM interaction and pseudo-dipole anisotropy by electric fields [20,21,22,23,24]; the DM interaction, which is known to induce the Doppler effect [18], similar to the STT [18,19], causes an asymmetric modification of the SW dispersion relation, where the SW frequencies are linearly shifted in the wavevector *k* direction by adding or subtracting the SW frequency for favored or unfavored chirality, respectively [25,26].

**Figure 1:**
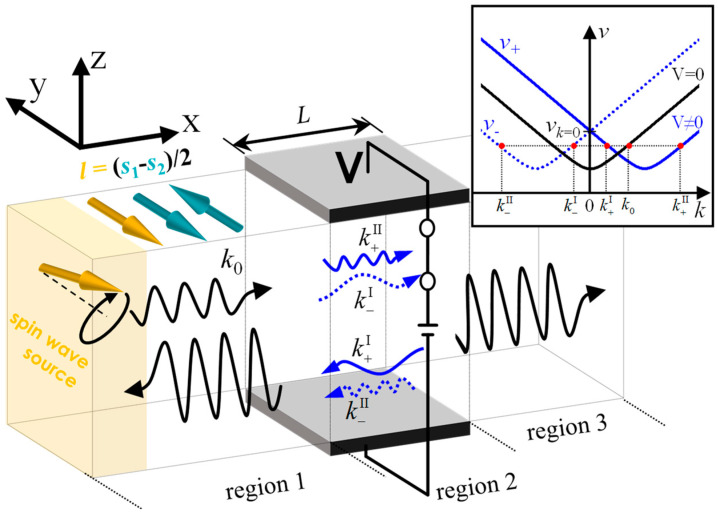
Schematics for antiferromagnetic spin wave (SW) propagation under the non-uniform electric field. The electric field induces the Dzyaloshinskii-Moriya (DM) interaction and pseudo-dipole anisotropy where the DM vector and easy direction of anisotropy are along *y* axis. Thus, chiral-dependent spin wave dispersion bands are formed (inset), where + and − indicate the right-handed and left-handed circularly polarized waves (blue solid line and dotted line), respectively, in region 2. The original band without the electric field is described as the black line. When the SWs propagate from region 1 to region 2, it is split into two waves, with $k_{+}^{II}$ and $k_{-}^{I}$ in region 2. In the resonant condition (*k*^I^ − *k*^II^)*L* = *nπ*, transmission T = |*t*|^2^, and R = |*r*|^2^ are dramatically enhanced in region 1 and region 3.

The proposed device setup is shown in Figure 1. Antiferromagnetic- and ferromagnetic-order parameters are defined as Néel order, **l** = (**s_i_** − **s_j_**)/2 and **m** = (**s_i_** + **s_j_**)/2, where each spin is normalized by its magnitude **s**_i_ = **S_i_**/|**S_i_**|. We generate SWs from the fluctuation of **l** at the left side, as indicated in the yellow box of Figure 1. The wire length is set to be long enough so that the SW is not reflected from the end of the wire. The inset of Figure 1 shows SW dispersion relations without and with DM interaction and pseudo-dipole anisotropy. The dispersion relations (or a graph of frequency v against wavevector *k*) relying on different chiralities (+ and − are right- and left-handed circularly polarized SW modes, respectively) are separated into two frequencies $v_{+}(k_{+})$ (solid blue line) and $v_{-}(k_{-})$ (dotted blue line) when the DM interaction is turned on, but they degenerate without the DM interaction (black line). The SWs with the wavevector, *k*, with the positive sign, propagate along the waveguide from region 1 to region 3. When the electric field is applied in region 2, four emerging SWs, $k_{-}^{I}$, $k_{+}^{II}$, $k_{-}^{II}$, and $k_{+}^{I}$ for $v<v_{k=0}$, are partially reflected and transmitted at both boundaries, and they are amplified in the resonance condition when (*k*^I^ − *k*^II^)*L*/*π* is an odd integer. Here, the role of the pseudo-dipole anisotropy is to secure the parabolic-like SW band by dragging SW bands upward in the presence of the DM interaction (see the inset of Figure 1). For $v<v_{k=0}$, the phase difference between the SWs is proportional to v, leading to the generation of multiple resonant modes, but for $v>v_{0}$, the phase difference between two chiral SWs is almost linear to v, and a single resonant mode is possibly generated. Due to the pseudo-dipole anisotropy, the bound state is prevented throughout the wire; in the bound state, the propagating SWs are subject to decay or tunneling [27,28].

## 2. Results

The exchange energy *J* has a negative sign for AFMs, and the magnetic crystalline anisotropy, *K*_y_, is positive along the *y* axis. AFM texture is along the long *x* axis of the wire and sandwiched by two electrodes with a length of *L,* and the voltage is applied between two electrodes. The geometric inversion asymmetry may induce the DM interaction along the *y* axis, according to **D_ij_** ∝ **z** × **e_ij_**, where the *z* axis is normal to the interface, and **e**_ij_ is the unit vector connecting neighbor spins **s**_i_ and **s**_j_ [20,29]. We ignore the geometric DM interaction to describe our scenario explicitly. Instead, when the electric field along the *z* axis breaks the inversion symmetry, the electric-field-induced DM vector, **D*_E_***, moves effectively toward the *y* axis due to **D**_ij_ ∝ *E*_z_**z** × **e**_ij_ [20,21,22,23,24]. When |**D_E_**| is strongly induced, the pseudo-dipolar anisotropy energy *K*_E_ in the easy plane should be taken into account, which is proportional to *E*_z_^1/2^ [20,21,22,23,24]. Here, the *y* component of *K*_E_ is considered, assuming that the AFM wire is a one-dimensional texture.

Under an exchange approximation where |*J|*≫*D*_y_, *K*_y_, and *K*_E_, we can assume that the magnetic moments are linearly distributed in space, $(l_{i+1}-l_{i})/d\sim l\prime =dl/dx$ and $(m_{i+1}-m_{i})/d\sim m\prime =dm/dx$, where *d* is the interspacing of the nearest neighbor Néel spins. The total energy *E*_1D_ is set as

(1) $$E_{1D}=a/2|m{|}^{2}+A/2|l\prime {|}^{2}+B(m\cdot l\prime -l\cdot m\prime )-\frac{K_{\mathrm{eff}\;}}{2}{\left(l\cdot y\right)}^{2}+\frac{{\tilde{D}}_{y}}{2}y\cdot (l\times l\prime ),$$

where *a*, *A,* and *B* are the homogeneous, the inhomogeneous, and the parity-breaking exchange constant, respectively [30]. These parameters are defined as $A=d^{2}J=J$, a=4J, B=dJ=J, and ${\tilde{D}}_{y}=dD_{y}=D_{y}$, where *d* is used as the unit length. The effective anisotropy, *K*_eff_, is defined as the summation of the pseudo-dipole anisotropy, *K*_E_, and crystalline anisotropy, *K*_y_. The Landau–Lifshitz–Gilbert (LLG) equations on **m** and **l** are derived from Equation (1):

(2a) $$\dot{l}=(\omega_{m}-\beta \dot{m})\times l,$$

(2b) $$\dot{m}=(\omega_{l}-\beta \dot{l})\times l,$$

where the effective magnetic fields $\omega_{m}/\gamma =h_{\mathrm{eff},m}$ and $\omega_{l}/\gamma =h_{\mathrm{eff},l}$ are defined as the functional derivative of energy density: $h_{eff,m}=-\partial E_{1D}/\partial m=-am-Bl\prime$ and $h_{eff,l}=\partial E_{1D}/\partial l=Al\prime \prime +Bm\prime +K_{z}l_{z}z+l\prime \times D$, respectively [30]. Here, we ignore the phenomenological damping constant, *β*, reflecting the property of an insulator.

**Figure 2:**
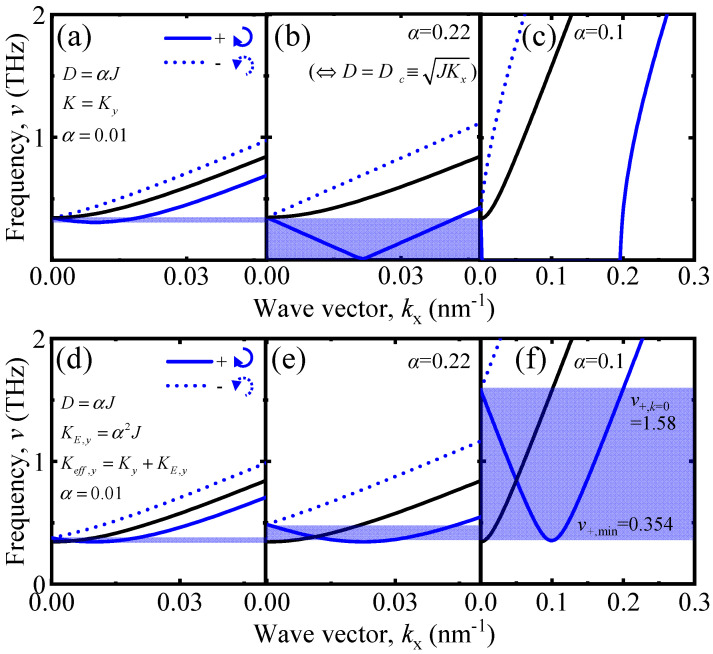
The spin wave (SW) dispersion relations in regions 1, 2, and 3 and the effective SW band for scattering, indicated by blue box. (**a**–**c**) SW dispersion relations as a function of the Dzyaloshinskii-Moriya (DM) interaction. (**d**–**f**) SW dispersion relations as a function of the voltage-induced DM interaction and pseudo-dipole anisotropy. Those interaction energies are characterized proportional to *α*. In region 2, two SWs consisting of left-handed circularly polarized wave (−, dotted blue line) and right-handed circularly polarized wave (+, solid blue line) bands are lifted. In the case of (**a**–**c**), the DM interaction makes the SW band shift downward, and the effective bandwidth becomes maximized at *D* = *D*_c_ and disappears for *D* >> *D*_c_. However, due to the pseudo-dipole anisotropy that adds an extra frequency to the SW band, the SW band is prevented from being below v=0.

By taking the cross product of **l** in Equation (2a), we obtained the analytical relation between **m** and **l**: $m=\dot{l}\times l/(a\gamma )$. Setting the first order for small excitations on $l\sim e_{r}+[l_{\theta }(x,t)e_{\theta }+l_{\varphi }(x,t)e_{\varphi }]$ in a spherical coordinate system and inserting ***m*** into Equation (2b), the equation of motion for SW excitations on $l_{\varphi }$ and $l_{\theta }$ are obtained: ${\ddot{l}}_{\varphi (\theta )}=(a\gamma^{2})(Al_{\varphi (\theta )}^{\prime \prime }-K_{y}l_{\varphi (\theta )}\pm D_{y}l_{\theta (\varphi )}^{\prime }),$ where the ± sign indicates the SW chirality. Using a plane wave ansatz $\psi_{\pm }\equiv l_{\theta }\pm il_{\varphi }\sim \left(\begin{array}{c} 1\\ \pm i \end{array}\right)e^{i2\pi v_{\pm }t}$, it results in the time-independent Schrödinger-type wave equations [31] and the non-degenerate SW dispersion relations of AFM:

(3) $${v_{\pm }}^{2}=(a{\gamma_{0}}^{2})[k^{2}A+K_{eff,y}\pm 2kD_{y}]$$

where the reduced gyromagnetic ratio $\gamma_{0}=\gamma /(2\pi )$ is used. The parameters suitable for antiferromagnetic insulators are set as *J* = −31.9 meV and *K*_z_ = 0.0005|*J*|. With these parameters, the SW dispersion relations for AFM are plotted in Figure 2a–c as a function of the DM interaction and Figure 2d–f as functions of the DM interaction and pseudo-dipole anisotropy; since it shows a symmetric dispersion relation for v, as in Equation (3), we omitted $v_{-}(k_{-})$ in Figure 2.

When |*D*_E_| and *K*_E_ are induced by the electric field [20,21,22,23,24], they are simply formulated with respect to *J*, $D_{E}=\alpha |J|$, and $K_{E}=\alpha^{2}|J|$, where *α* is proportional to the electric field; even in a strong *D*_E_, the chiral structure is suppressed by *K*_E_ because the instability condition is defined as $D_{\mathrm{chiral}}=\sqrt{2JK_{\mathrm{eff},y}}$.

The effective SW band with + chirality for *k* > 0 (with − chirality for *k* < 0) is defined as the frequency range from $v_{+,\min}$ to $v_{+,k=0}=v_{k=0}$ (from $v_{-,\min}$ to $v_{-,k=0}$ in Figure 1), as indicated by the blue box in Figure 2, where $v_{\pm ,k=0}$ is the frequency at *k* = 0, and $v_{\pm ,\min}$ is the minimum frequency.

Consider the SW dispersion relation relying on DM interaction. In Figure 2a,b, the effective SW band width is proportional to *D* in a finite range: $0<D\leq D_{c}$ or 0<α≤0.22 and starts to decrease for *D* ≥ *D*_c_ because $v_{+}$ and $v_{-}$ become partially imaginary. In the end, the effective SW band disappears as $D\gg D_{c}$ or *α* = 0.1, where the phase difference between two SWs is nearly constant (see Figure 2c). In all cases above, SWs within the effective SW band could not transmit into region 2 because SWs in region 2 belong to the forbidden band of region 1 (see Figure 2a,b), where the SW tunneling phenomenon is excluded [25,26].

However, as shown in Figure 2d–f, when the pseudo-dipole anisotropy is included, the effective SW band in region 2 overlaps with the degenerate band of region 1 for all ranges of *α* because the pseudo-dipole anisotropy energy adds frequencies of chiral SW modes. In the ferromagnetic system, the SW dispersion relation is obtained as ${\left(v\pm 2D_{y}\sin[ak]\right)}^{2}={\left(2J+K_{y}-2J\cos[ak]\right)}^{2}$ by replacing the sign of *J* with a positive. Here, the spin wave frequency is not imaginary under the strong DM interaction. Instead, the negative spin wave band becomes partially positive, and corresponding SWs undergo scattering with each other [18].

Now, we solve the SW scattering problem using the transfer matrix method (TMM) [32]. The TMM is an excellent tool for solving the linearized wave equation; the SW dispersion relation without linear approximation is obtained as ${v_{\pm }}^{2}={\left(2J+K_{\mathrm{eff}}\right)}^{2}-4{\left(J\cos[ak]\pm D\sin[ak]\right)}^{2}$, and it is almost identical to Equation (3) for *α* = 0.1. For *α* > 0.1, another approach to calculate the scattering problem is necessary for the non-linear Schrödinger equation.

At first, we consider wavefunctions $\psi_{\pm }^{II}$ with a single wavevector $k^{II}=|k_{-}^{II}|=|k_{+}^{II}|$ at two points, *x*_a_ and *x*_b_: $k_{+}^{II}$ for forward propagation and $k_{-}^{II}$ for backward propagation (see the inset of Figure 1). When the polarization of incident SWs is parallel to the *z* axis (see Figure 1), the phase differences between $\psi_{+}^{II}$ and $\psi_{-}^{II}$ with respect to $l_{z}(=\psi_{+}^{II}+\psi_{-}^{II})$ and $l_{x}(=\psi_{+}^{II}-\psi_{-}^{II})$ are 0 and *π*, respectively. They are decomposed into two complex wave components, $\psi_{\pm }^{II}(x_{a})$ and $\psi_{\pm }^{II}(x_{b})$. Since the two functions $\psi_{\pm }^{II}$ are required to be continuous [18,32], they are connected by inner products with two matrix components; the one is related to the phase evolution across a constant potential, $M_{p}^{II}=\left(\begin{array}{cc} e^{jk_{i}^{II}l} & 0\\ 0 & (\pm )e^{-jk_{i}^{II}l} \end{array}\right)$, and the other is the transfer matrix on the potential step,

$$M_{s}^{II}(k_{i}^{II},k_{i+1}^{II})=\frac{1}{2}\left(\begin{array}{cc} 1+k_{i}^{II}/k_{i+1}^{II} & \left(\pm )(1-k_{i}^{II}/k_{i+1}^{II}\right)\\ \left(\pm )(1-k_{i}^{II}/k_{i+1}^{II}\right) & 1+k_{i}^{II}/k_{i+1}^{II} \end{array}\right)$$

The upper (the lower) one of (±) signs is attributed to the phase difference between $\psi_{+}^{II}$ and $\psi_{-}^{II}$ for *l*_z_ (*l*_x_); for example, $e^{i0}=+1$ ($e^{i\pi }=-1$). When the incident SWs are linearly polarized along the *x* axis, the upper and the lower signs should be reversed.

Overall transfer matrices from *x* = 0 to *x* = *x*_0_ are expressed as

$$\left(\begin{array}{c} 0\\ t^{II}(x_{0}) \end{array}\right)=M^{II}\left(\begin{array}{c} 1\\ r^{II}(x_{0}) \end{array}\right)$$

and

$$M^{II}=M_{p}^{II}(k_{i=N}^{II},l)M_{s}^{II}(k_{i=N}^{II},k_{i=N-1}^{II})\cdots M_{p}^{II}(k_{i=1}^{II},l)M_{s}^{II}(k_{i=1}^{II},k_{i=0}^{II})M_{p}^{II}$$

where *x*_0_ is divided into *N* segments with uniform spacing; *l* and *i* is the segment number. Therefore, *t*(*x*_0_) and *r*(*x*_0_) are defined as det(*M*)/*M*_22_ and *M*_21_/*M*_22_, respectively, where det is the determinant of the matrix, and *M*_ij_ is denoted by the element at row *i* and column *j* [32].

Now, wavefunctions with two different *k* are taken into account: $k_{+}^{II}$ and $k_{-}^{I}$ for forward propagation and $k_{-}^{II}$ and $k_{+}^{I}$ for backward propagation (see the inset of Figure 1). Since $|k_{-}^{I}|=|k_{+}^{I}|=k^{I}$ and $|k_{-}^{II}|=|k_{+}^{II}|=k^{II}$, two different wavevectors $k^{I}$ and $k^{II}$ are derived as an inverse function of v(k):

$$k^{I}(v)=\frac{D_{y}}{A}-\sqrt{{\left(\frac{D_{y}}{A}\right)}^{2}-\frac{K_{\mathrm{eff}}}{A}+\frac{v}{Aa\gamma_{0}^{2}}}$$

and

$$k^{II}=\frac{D_{y}}{A}+\sqrt{{\left(\frac{D_{y}}{A}\right)}^{2}-\frac{K_{\mathrm{eff}}}{A}+\frac{v_{\pm }^{2}}{Aa\gamma_{0}^{2}}}$$

Therefore, wave components are divided by two folds as $\psi_{b}^{I}=M^{I}\psi_{a}^{I}$ and $\psi_{b}^{II}=M^{II}\psi_{a}^{II}$. Thus, the total matrix is recast into

$$\begin{array}{l} M^{III}=\left(0.5M_{p}(k_{i=N}^{I},l)\cdot M_{s}(k_{i=N}^{I},k_{i=N-1}^{I})+0.5M_{p}(k_{i=N}^{II},l)\cdot M_{s}(k_{i=N}^{II},k_{i=N-1}^{II})\right)\cdots \\ \left(0.5M_{p}(k_{i=2}^{I},l)\cdot M_{s}(k_{i=1}^{I},k_{i=2}^{I})+0.5M_{p}(k_{i=2}^{II},l)\cdot M_{s}(k_{i=1}^{II},k_{i=2}^{II})\right) \end{array}$$

where $\psi_{b}^{III}=M^{III}\psi_{a}^{III}$. Therefore, *t*^III^(*x*_0_) and *r*^III^(*x*_0_) are obtained from *M*^III^, respectively.

**Figure 3:**
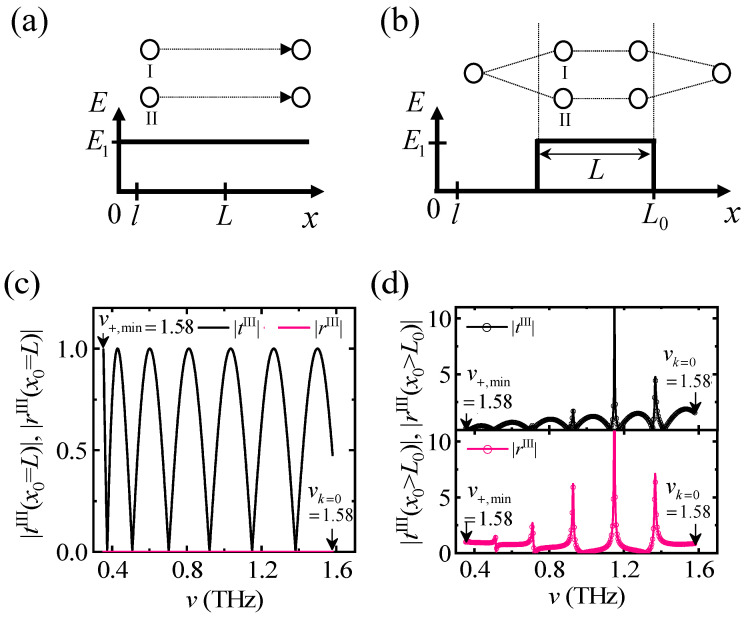
Two types of electric field distribution and corresponding transmission coefficient *t* and reflection coefficient *r* calculated from the frequency range from $v=v_{\pm ,\min}$ to $v=v_{k=0}$ for $l_{\theta }$. (**a**) Uniform electric field distribution. (**b**) Non-uniform electric field distribution. (**c**) *t* and *r* for the uniform case. (**d**) *t* and *r* for the non-uniform case. In (**a**), two SWs with different wavevectors *k*^I^ and *k*^II^ propagate without reflection, but in (**b**), the degenerate SW is split into two SWs in region 2, where two reflection channels at boundaries are activated due to *k* change and play the role of a resonant cavity. Here, the results for $l_{\theta }$ are identical to those for $l_{\varphi }$.

Figure 3 shows two types of electric field profiles and corresponding *t*^III^(*x*_0_) and *r*^III^(*x*_0_) calculated from $v=v_{\pm ,\min}$ to $v=v_{k=0}$ for *l*_z_. First, consider *t*^III^(*x*_0_ = *L*) and *r*^III^(*x*_0_ = *L*) for the electric field applied uniformly throughout the wire in Figure 3a,c. The electric field, *E*_1_ induces the DM interaction and pseudo-dipole anisotropy that corresponds to *α =* 0.1, as shown in Figure 2f. *M*_s_ is the identity matrix because the off-diagonal component of *M*_s_ is zero, and the diagonal components of *M*_0_ are not coupled with each other; for example, $M_{p}(k^{I},l)\cdot \cdot \cdot M_{p}(k^{I},l)=M_{p}{\left(k^{I},l\right)}^{N}=M_{p}(k^{I},Nl)=M_{p}(k^{I},L)$, and thereby, the transfer matrix is summarized as $M^{III}=0.5M_{p}(k^{I},L)+0.5M_{p}(k^{II},L)$. As a result, $|r^{III}(x_{0}=L)|=0$ and $\left|t^{III}(x_{0}=L)|=|\cos\Delta_{rel}\right|$ lead to oscillating patterns with the relative phase difference, $\Delta_{rel}=(k^{I}-k^{II})L$, as the constructive (destructive) interference occurs when $\Delta_{rel}/\pi$ is an even (odd) integer, respectively. In addition, the oscillating period is not constant because of parabolic-like SW dispersion relation of AFM, as shown in Figure 3c. The number of oscillations is proportional to *L* because a number of SWs that take part in the interference is proportional to *L*; in small *L*, SWs with low *k* could not achieve sufficient phase evolution for the interference.

Second, consider *t*^III^(*x*_0_ > *L*_0_) and *r*^III^(*x*_0_ > *L*_0_) when the electric field distribution is given as a barrier-like function in Figure 3b. *M*_s_ is not an identity matrix, and the reflection channels are activated at boundaries. *M*^III^ is simplified as $M^{III}=0.5M_{s}(k_{E_{1}}^{I},k_{0})M_{p}(k^{I},L)M_{s}(k_{0},k^{I})+0.5M_{s}(k^{II},k_{0})M_{p}(k^{II},L)M_{s}(k_{0},k^{II})$, where $M_{s}(k^{I(II)},k_{0})$ and $M_{s}(k_{0},k^{I(II)})$ are the matrices at boundaries. It is confirmed that the numerical result is identical to the analytically simplified equation (see open circles and solid line in Figure 3d). It suggests that incoming and outgoing SWs outside the cavity have a negligible contribution to amplification. As a result, *t*^III^(*x*_0_ > *L*_0_) and *r*^III^(*x*_0_ > *L*_0_) are calculated as

$$|t^{III}(x_{0}>L_{0})|=\left|\frac{\left(ik_{0}\left(2+\cos(2(k^{II}-k^{I})L)+\cos(2(k^{II}+k^{I})L)\right)k^{I}k^{II}+(k^{I2}+k^{II2})\sin(2k^{I}L)\sin(2k^{II}L)\right)}{2i\left(\cos(2k^{I}L)+\cos(2k^{II}L)\right)k_{0}k^{II}k^{I}+({k_{0}}^{2}+k^{I2})k_{E_{1}}^{+}\sin(2k^{I}L)+({k_{0}}^{2}+k^{II2})k^{I}\sin(2k^{II}L)}\right|$$

and

(4) $$|r^{III}(x_{0}>L_{0})|=\left|\frac{-k_{0}^{2}\left(k^{II}\sin(2k^{I}L)+k^{I}\sin(2k^{II}L)\right)+k^{I}k^{II}\left(k^{II}\sin(2k_{E_{1}}^{II}L)+k^{I}\sin(2k^{I}L)\right)}{2i\left(\cos(2k^{I}L)+\cos(2k^{II}L)\right)k_{E_{0}}k^{II}k^{I}+({k_{0}}^{2}+k^{I2})k^{II}\sin(2k^{I}L)+({k_{0}}^{2}+k^{II2})k^{I}\sin(2k^{II}L)}\right|.$$

**Figure 4:**
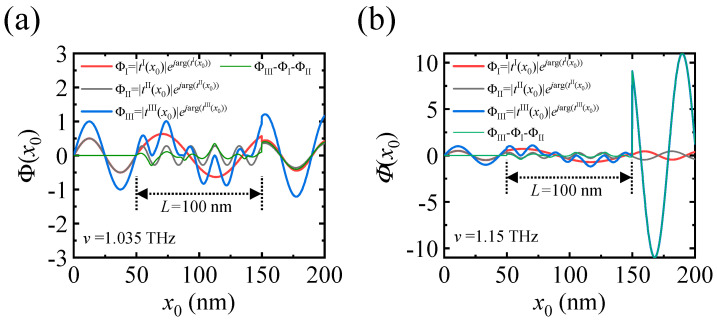
Spatially resolved spin wave (SW) propagation profiles using the propagating parameters, *Φ*^I^, *Φ*^II^, *Φ*^III^, and *Φ*^III^ − *Φ*^I^ − *Φ*^II^. (**a**) In-phase condition where (*k*^I^ − *k*^II^)*L*/*π* = 8. (**b**) Out-of-phase condition where (*k*^I^ − *k*^II^)*L/π* = 9. When *Φ*^I^ and *Φ*^II^ are in-phase, all SW components are added up to maximize |*Φ*^III^|, resulting in |*t*^III^| > 1. However, SWs with out-of-phase conditions give rise to the resonant amplification ($|t^{III}|\gg 1$) due to the SW scattering effect.

Here, |*t*^III^(*x*_0_ > *L*_0_) | and |*r*^III^(*x*_0_ > *L*_0_)| curves are characterized with oscillating patterns and resonant peaks (see Figure 3d); the oscillating maxima occur when $\Delta_{rel}/\pi$ is an even integer, which corresponds to the case in which two SWs have an in-phase condition. The resonant amplification takes place at the frequency where $\Delta_{rel}/\pi$ is an odd integer or two SWs are of an out-of-phase condition (see Figure 4b).

To understand SW spectroscopy explicitly, the spatially resolved SW propagation profiles are calculated using the propagating parameters, *Φ*^III^(*x*_0_) = |*t*^III^(*x*_0_)|exp(*i*arg(*t*^III^(*x*_0_))) for *v*_+_ = 1.035 THz and 1.15 THz (see Figure 4a,b), together with *Φ*^I^(*x*_0_) = |*t*^I^(*x*_0_)|exp(*i*arg(*t*^I^(*x*_0_))) and *Φ*^II^(*x*_0_) = |*t*^II^(*x*_0_)|exp(*i*arg(*t*^II^(*x*_0_))). Here, arg(*t*(*x*_0_)) indicates the transmission phase. In *v*_+_ = 1.035 THz, $\Delta_{rel}/\pi$ = 8 is an even number, indicating an in-phase condition between *Φ*^I^(*x*_0_) and *Φ*^II^(*x*_0_). However, both *Φ*^I^(*x*_0_) and *Φ*^II^(*x*_0_) have a non-resonant condition for the cavity, *k*^I^*L*/*π* = 10.34 and *k*^II^L/*π* = 2.34. If they are resonant with the cavity where both *k*^I^*L*/*π* and *k*^II^*L*/*π* are even integers, they propagate with |*t*(*x*_0_ > 150 nm)| = 1, |*r*(*x*_0_ > 150 nm)| = 0. Until *x*_0_ = 50 nm, spin wave propagation is similar to the non-scattering case because *M*_s_ is an identical matrix. Therefore, *Φ*^I^(*x*_0_) and *Φ*^II^(*x*_0_) are in-phase, and their summation is identical to *Φ*^III^(*x*_0_), as shown in Figure 4a. For 50 nm < *x*_0_ < 150 nm, a single SW with *k*_0_ is split into two SWs with *k*^I^ and *k*^II^. They undergo the reflection and the transmission at *x* = 50 nm. As a result, |*Φ*^I^(*x*_0_)| is larger because the wavevector *k*^I^ is lower than *k*_0_; in general, the transmission coefficient propagating from medium 1 to medium 2 is defined as 2/(1 + *k*_2_/*k*_1_). This relation explains the reduced amplitude of *Φ*^II^(*x*_0_), where *k*^II^ is larger than *k*_0_. Remarkably, *Φ*^III^(*x*_0_) can be larger than the summation of *Φ*^I^(*x*_0_) and *Φ*^II^(*x*_0_) because of the existence of SW scattering *Φ*^III^(*x*_0_) − (*Φ*^I^(*x*_0_) + *Φ*^II^(*x*_0_)). For *x*_0_ > 150 nm, two SWs experience transmission and reflection both at *x* = 50 nm and at *x* = 150 nm, where SW scattering plays an additional role in |*t*^III^(*x*_0_)|; |*t*^III^(*x*_0_)| can be larger than 1 (see Figure 3b). Even though two SWs are simultaneously resonant with the cavity, or |*t*^I^(*x*_0_)| = 0.5 and |*t*^II^(*x*_0_)| = 0.5, |*t*^I^(*x*_0_)| + |*t*^II^(*x*_0_)| cannot be larger than 1 without SW scattering.

Now, we discuss the out-of-phase condition of *v*_+_ = 1.15 THz where $\Delta_{rel}$/*π* = 9. At *x*_0_ > 150 nm, |*Φ*^III^| increases dramatically because SWs are resonant with each other; abrupt changes in amplitude at *x*_0_ = 50 nm and *x*_0_ = 150 nm are shown in Figure 4b. It proves again that *Φ*^III^ includes SW scattering by showing that *Φ*^III^ is identical to *Φ*^III^ − (*Φ*^I^ + *Φ*^II^) for *x*_0_ > 150 nm. For example, *Φ*^I^ and *Φ*^II^ are canceled out, and the contribution to the amplification is solely ascribed to two-SW resonance. Compared to SW resonance with the cavity, resonance between SWs is characterized by a distinct phase evolution pattern. When SW is resonant with the cavity, the phase shift is simply estimated by *nπ*, where *n* is an integer. The phase shift at a SW-scattering resonant frequency that depends on *L* is *k*^I^*L*/π + *nπ* or *k*^II^*L*/*π* + (*n +* 1)*π*. Similar to the case of the uniform electric field, the number of amplified SWs depends on *L*. The amplified SW frequency is tuned by the strength of the electric field because it modifies the SW band structure. It implies that the electric field plays the role of programmable SW modular for an incoming SW packet. Both in the uniform and non-uniform electric field, $\left|t^{III}\right|$ and $\left|r^{III}\right|$ on *l*_x_ and *l*_z_ are identical with each other, implying that when the SWs that polarized fully along the *x* or *z* axis are incident to region 2, the same resonant effect is expected to occur.

Finally, we attempt to estimate the strength of the electric field required for applications. Mott insulator AFM with transition metal compounds is known to have the characteristic spin-orbit coupling energy *E*_SO_ ~ 3 eV in Y_3_Fe_5_O_12_ garnet [21]. Therefore, the electric field required for *α* = 0.1 is roughly obtained from the relation $\left|E|=E_{SO}\alpha /(ed\right)$ ~ $0{.3\;\mathrm{Vnm}}^{-1}$, where *d* is the distance between the nearest neighbor magnetic ions and is set as ~1 nm [21]. In our research, we have primarily focused on the THz regime. However, our findings suggest that assuming the magnetic system is identical to the Mott insulating AFM, the amplification of spin waves is achievable even in the gigahertz frequency range (see Table 1).

Recent research on the manipulation of the polarization has focused on the control of relative phase between SWs with different chirality using non-collinear spin structures [6,7] or inhomogeneous DM interaction steps [33]. However, this work shows that the coherent scattering between two opposite chiral SWs triggers resonant amplification. Therefore, both the amplitude and polarization can be controlled by operating multiple gates in one medium if the electric field is properly controlled. As a result, the high-amplitude SW with controlled polarization would be realized for applications for various magnon devices.

**Table 1:** The comparison of our work with existing AFM materials.

	Our Work	YFeO_3_ [34]	NiO [35]	α-Fe_2_O_3_ [36]
*J*	−31.9 meV	−2.48 meV	−112.6 meV	−107.6 meV
*K*	0.15 meV (easy axis)	0.0115 meV (easy axis)	−0.072 meV (hard axis)	−0.002 meV (hard axis)
Resonance frequency	0.34 THz	0.299 THz (low-frequency mode) 0.52 THz (high frequency mode)	1 THz	0.148 THz (low-temperature phase)

## 3. Conclusions

In this work, we investigate two-SWs’ resonance and amplification under the uniform and non-uniform electric field profiles. Since the electric field induces the DM interaction and pseudo-dipole anisotropy simultaneously, SW bands are shifted laterally in the v-k space, and the broad effective SW band is secured. Apart from the uniform electric field distribution where the transmitted coefficient |*t*| oscillates with the phase evolution, $\Delta_{rel}=(k_{E_{1}}^{I}-k_{E_{1}}^{II})L$, the non-uniform electric field forms the reflection channel at both boundaries and, thus, a resonant cavity. SW amplification is triggered by virtue of the resonance effect between two SWs. Throughout the phase analysis for each SW propagation, we conclude that the scattering component between two SWs has the main contribution to the amplification.

## Data Availability

The original contributions presented in the study are included in the article, further inquiries can be directed to the corresponding author.
